# 

**Published:** 1837-01

**Authors:** 


### BRITISH AND FOREIGN

### MEDICAL REVIEW

OR

### QUARTERLY JOURNAL

OF

### PRACTICAL MEDICINE AND SURGERY

LIBRARY OF THE
Orleans Parish Medical Society

EDITED BY

JOHN FORBES M.D. F.R.S.

AND

JOHN CONOLLY M.D.

EDITORS OF THE CYCLOPÆDIA OF PRACTICAL MEDICINE

VOL. III

JANUARY—APRIL 1837

LONDON
SHERWOOD GILBERT AND PIPER
PATERNOSTER ROW
MDCCCXXXVII

LONDON:
PRINTED BY J. AND C. ADLARD, BARTHOLOMEW CLOSE.

### BOOKS RECEIVED FOR REVIEW.

ENGLISH.

1. Thoughts on Physical Education, and the true Mode of improving the Condition of Man; and on the Study of the Greek and Latin Languages. By Charles Caldwell, M.D., Professor of the Institutes of Medicine, &c. in Transylvania University. With Notes by Robert Cox, and a recommendatory Preface by Geo. Combe.—Edinburgh, 1836. 12mo. pp. 198. 3s. 6d.

2. Principles of Pathology and Practice of Physic. By John Mackintosh, M.D., Lecturer on the Practice of Physic in Edinburgh, &c. Fourth Edition.—London and Edinburgh, 1836. Two vols. 8vo. £1 11s.6d.

3. Observations on the Influence of Religion upon the Health and Physical Welfare of Mankind. By Amariah Brigham, M.D.—Boston, 1835. 8vo. pp. 301.

4. Remarks on the Influence of Mental Cultivation and Mental Excitement upon Health. By Amariah Brigham, M.D. Second Edition.—Boston, 1833. 8vo. pp. 130.

5. A Popular Manual of the Art of Preserving Health, embracing the Subjects of Diet, Air, Exercise, Gymnastics, general and physical Education, &c. &c. Designed for the use of all Ranks and Professions in Society. By J. B. Davis, Surgeon.—London, 1835. 8vo. 10s.

6. Tenth Report of the Connecticut Retreat for the Insane.—Hartford (U.S.), 1834. 8vo. pp. 21.

7. Facts and Cases in Obstetric Medicine, with Observations on some of the most important Diseases incidental to Females. By J. T. Ingleby, Surgeon to the Magdalen Asylum, and Lecturer on Midwifery at the Royal School of Medicine, Birmingham.—London, (no date.) 8vo. pp. 296. 9s.

8. The Transylvania Journal of Medicine and the associate Sciences. Edited by L. P. Yandell, M.D., Professor of Chemistry and Pharmacy in Transylvania University. For January, February, and March, 1836. —Lexington. 8vo. pp. 200.

9. The Western Journal of the Medical and Physical Sciences. Edited by D. Drake, M.D., Professor of Medicine in Cincinnati College, and W. Wood, M.D. April, 1836. —Cincinnati. 8vo.

10. A Treatise on Tetanus; being the Essay for which the Jacksonian Prize for the year 1834 was awarded by the Royal College of Surgeons in London. By T. B. Curling, Assistant Surgeon to the London Hospital.—London, 1836. 8vo. pp. 236. 8s.

11. The Clinique Médicale; or, Reports of Medical Cases. By G. Andral, &c. &c. Translated by D. Spillan, M.D. Part V.—London, 1836. 8vo. 5s.

12. Practical Observations on Strangulated Hernia, and some of the Diseases of the Urinary Organs. By Joseph Parrish, M.D.—Philadelphia, 1836. 8vo. pp. 330; with three Plates.

13. An Account of the most frequented Watering Places on the Continent, and on the Medicinal Application of their Mineral Springs; with Tables of Analysis, and an Appendix on English Mineral Waters. By Edwin Lee, Esq. M.R.C.S.—London, 1836. 8vo. pp. 232.

14. A Medical Vocabulary; or Explanation of all Names, Synonymes, Terms, and Phrases used in Medicine, Surgery, and the relative Branches of Medical Science; giving the correct Derivation, and Directions for the proper Pronunciation of each. By a Medical Practitioner.—Edinburgh, 1836. 12mo. pp. 185. 4s. 6d.

15. The Human Brain, its Configuration, Structure, Development, and Physiology: illustrated by References to the Nervous System in the lower Orders of Animals. By Samuel Solly, Lecturer on Anatomy and Physiology in St. Thomas's Hospital.—London, 1836. 8vo. pp. 492; with 12 Plates. 12s. 6d.

16. Guy's Hospital Reports, No. III. September, 1836. Edited by G. H. Barlow, M.A., and J. P. Babington, M.A.—8vo., with numerous Plates. 4s.

17. On the Disease of the Hip-Joint. By William Coulson, Consulting Surgeon to the London Lying-in Hospital, &c. &c. —London, 1837. 4to. pp. 111; with plain and coloured Plates. 10s. 6d.

18. A Bedside Manual of Physical Diagnosis, applied to Diseases of the Lungs, Pleura, Heart, Vessels, Abdominal Viscera, and Uterus. By Charles Cowan, M.D. P. & E. &c.—London, 1836. 18mo. pp. 58. 2s. 6d.

19. A Practical Demonstration of the Human Skeleton. By George Elkington, M.R.C.S., Demonstrator of Anatomy in the Royal School of Medicine and Surgery, Birmingham.—London and Birmingham, 1836. 12mo. pp. 240. 7s.

20. An Essay on the Origin and Nature of Tuberculous and Cancerous Diseases. Read to the Medical Section of the British Association, on the 23d August, 1836. By Richard Carmichael, M.R.I.A., Consulting Surgeon of the Richmond Surgical Hospital, &c.—Dublin, 1836. 8vo. pp. 56.

21. Medical Study: an Introductory Address, delivered at the Bristol Medical School, October 1, 1836, at the Opening of the Winter Session. By J. A. Symonds, M.D., Physician to the Bristol General Hospital, and Lecturer on the Theory and Practice of Medicine.—Bristol, 1836. 8vo. pp. 34. 1s.

22. A Theoretical and Practical Treatise upon the Ligature of Arteries. Translated from the French of P. J. Manec, M.D. &c., by J. W. Garlick, M.R.C.S., and W. C. Copperthwaite, M.R.C.S. With Notes and Appendices.—Halifax, 1832. 4to. pp. 227; with coloured Plates.

23. Selections from the Phrenological Journal: comprising forty Articles in the first five Volumes, chiefly by George Combe, James Simpson, and Dr. Andrew Combe. Edited by Robert Cox.—Edinburgh, 1836. 8vo. pp. 360. 5s. 6d.

24. The Principles and Practice of Obstetric Medicine, in a Series of Systematic Dissertations on Midwifery and on the Diseases of Women and Children. Illustrated by numerous Plates. By D. D. Davis, M.D. M.R.S.L., Professor of Midwifery in the University of London, &c.—London, 1836. Two vols. 4to. pp. 1294. £4 4s.

25. Animal Magnetism and Homœopathy; being the Appendix to Observations on the Medical Institutions, &c. of France, Italy, and Germany. By E. Lee, M.R.C.S. —London, 1835. 8vo. pp. 40. 2s.

26. Outlines of a Course of Lectures on Medical Jurisprudence. By T. S. Traill, M.D., Professor of Medical Jurisprudence in the University of Edinburgh, &c. &c.—Edinburgh, 1836. Small 8vo. pp. 94. 2s. 6d.

27. The British Medical Almanack for 1837. Edited by William Farr. To be continued annually.—London, 1837. Small 8vo. pp. 214. 3s.

28. Medical Report of the Managers of the Lunatic Asylum of Aberdeen. Read at the General Meeting, 9th June, 1836.—Aberdeen, 1836. pp. 12.

29. The Fallacy of the Art of Physic as taught in the Schools; with the Development of new and important Principles of Practice. By Samuel Dickson, M.D., Cheltenham, formerly a Medical Officer on the Staff.—Edinb. 1836. 8vo. pp. 180. 7s.

30. Homœopathy examined; or, Homœopathy in Theory, Allopathy in Practice. By R. Verity, M.D.—Paris, 1836. 8vo. pp. 24.

31. Essay on the Mineral Waters of Carlsbad, for Physicians and Patients. By Chevalier John De Carro, M.D. of the Faculties of Edinburgh, Vienna, and Prague, &c.—Prague, 1835. 12mo. pp. 136.

32. Lectures on the Morbid Anatomy of the Serous and Mucous Membranes. In two Volumes. Vol. I. of the Serous Membranes; and, as appended Subjects, Parasitical Animals, Malignant adventitious Structures, and the Indications afforded by Colour. By Thomas Hodgkin, M.D., Demonstrator of Morbid Anatomy, and Curator of the Museum at Guy's Hospital, &c. &c. —London, 1836. 8vo. pp. 402. 10s. 6d.

33. Elements of the Practice of Physic; presenting a View of the present State of Special Pathology and Therapeutics. By

David Craigie, M.D. F.R.S.E., Physician to the Royal Infirmary, Edinburgh, &c. &c. Vol. I.—Edinburgh, 1836. 8vo. pp. 952. 18s.

34. A Practical Treatise on the Management and Diseases of Children. By R. T. Evanson, M.D., Professor of Medicine, and H. Maunsell, M.D., Professor of Midwifery in the Royal College of Surgeons in Ireland. —Dublin, 1836. 8vo. pp. 527. 7s. 6d.

35. The Retrospective Address upon Medical Science and Literature, delivered at the Fourth Anniversary Meeting of the Provincial Medical and Surgical Association, held at Manchester, July 21st, 1836. By John Green Crosse, Esq. F.R.S., Surgeon to the Norfolk and Norwich Hospital. From the Transactions of the Provincial Medical and Surgical Association.—8vo. pp. 88. Worcester, 1836.

36. Catalogue Raisonné; or, classified Arrangement of the Books in the Library of the Medical Society of Edinburgh, instituted 1737.—Edinburgh, 1837. 8vo. pp. 342.

37. The Principles of Surgery. By James Syme, F.R.S.E., Professor of Clinical Surgery in the University of Edinburgh.—8vo. pp. 460. Edinburgh, 1837.

38. Elements of Medicine. Vol. I. On Morbid Poisons. By Robert Williams, M.D. Senior Physician of St. Thomas's Hospital. —London, 1836. 8vo. pp. 342. 10s. 6d.

39. A new and familiar Treatise on the Structure of the Ear and on Deafness. By A. W. Webster.—London, 1836. 8vo. pp. 151. 5s.

40. Micrographia: containing Practical Essays on Reflecting, Solar, Oxyhydrogen Gas Microscopes, &c. By C. R. Goring, M.D., and A. Pritchard, Esq.—London, 1837. 8vo. pp. 231. 8s. 6d.

41. Experimental Researches into the Physiology of the Human Voice. By John Bishop, M.R.C.S. &c.—London, 1836. pp. 24; plates. 2s.

42. A Description of the Bones; together with their several connexions with each other, and with the muscles, specially adapted for Students in Anatomy. By J. F. South, Assistant Surgeon to St. Thomas's Hospital.—Third Edition, with 250 Engravings by Branston. 7s.

FOREIGN.

1. Der torpide Croup, die gefahrvolleste Art der häutigen Bräune. Ein Beitrag zur nähern Erforschung der natur des Croups, zur Diagnostik und glucklichen heilmethode der verschiedenen arten und zu einer neuen theorie desselben. Von Philipp von Hagen, M.D., mit zusatzen, &c. über das wesen des Torpors, &c. Von L. A. Kraus, Dr. Phil. et M. leg., &c.—Göttingen, 1835. 8vo. pp. 220.

2. Lehrbuch der Geburtshülfe fur Hebammen. Von F. K. Nägele, M.D., Professor der Medizin und Geburtshülfe zu Heidelberg, &c. Dritte Auflage.—8vo. pp. 406. Heidelberg, 1836.

3. Katechismus der Hebammenkunst, als anhang zu seinem Lehrbuche, &c. Vom Dr. F. K. Nagele, &c. IIIe Auflage.—Heidelberg, 1836. 8vo. pp. 127.

4. Hygiène Publique; ou Memoires sur les Questions les plus importantes de l'Hygiène appliquée aux professions et aux travaux d'Utilité publique. Par A. J. B. Parent-Duchatelet, Médecin de l'Hôpital de la Pitie, &c.—Paris, 1836. Two vols. 8vo. pp. 552, 708.

5. De la Prostitution dans la Ville de Paris, considérée sous la Rapport de l'Hygiène publique, de la Morale et de l'Administration. Par A. J. B. Parent-Duchatelet, M.D. &c.—Paris, 1836. Two vols. 8vo.

6. Denkwürdigkeiten in der artzlichen praxis. Von Dr. J. H. Kopp, Kurfürstlich geheimen Ober-medicinalrathe, &c. &c. zu Hanau. Dritter Band.—Frankfurt, A.M. 1836. 8vo. pp. 407.

7. Specimen Historico-Medicum, de Choleræ Asiaticæ itinere per Belgium Septentrionale. Auctore A. C. G. Suerman, M.D.—Trajecti ad Rhenum, 1835. 8vo. pp. 289.

8. Rede zur Feier des zwei und vierzigsten Stiftungstags des Königlichen Medicinisch-chirurgischen Friedrich-Wilhelms-Instituts, am 2ten August, 1836, gehalten von Dr. Johannes Müller, Professor der Anatomie und Physiologie.—Berlin. 8vo. pp. 27.

9. Ueber die Endungsweise der Nerven in den Muskeln, nach einigen untersuchungen. Von Dr. Fr. Carl Emmert, privatdocenten an der Hochschule in Bern.—Bern, 1836. 4to. pp. 35; mit 2 Abbildungen.

10. Beschreibung der St. Petersburgischen Anstalt zur bereitung künstlicher mineralwässer sowohl zum trinken als zum baden. Von Dr. M. A. Meyer. 8vo. pp. 64. St. Petersburg, 1834.—Bericht ueber die Wirksamkeit der St. Petersburgischen Anstalt, &c. im Jahre, 1836. 8vo. pp. 12. St. Petersburg, 1836.

11. Effet des Eaux de Carlsbad sur les derniers Fragmens d'un Calcul restés dans la Vessie, après la Lithotripsie. Lettre du Docteur Bigel au Chevalier De Carro.—Prague, 1836. 8vo. pp. 24.

12. Il Filiatre-Sebezio Giornale delle Scienze Mediche. Diretto dal Prof. S. M. Ronchi, Compilato da Salvatore de Renzi. Anno VI. Vol. XI.—Napoli, 1836.

13. Bullettino delle Scienze Mediche. Serie 2a. Vol. II.—Bologna, 1836.

14. Texture et Developpement des Poumons. These Soutenue 24 Juin, 1836. Par M. A. Berard, Chirurgien de l'Hôpital Necker.—Paris, 1836. 8vo. pp. 132.

